# Durability Optimization of Fiber Grating Hydrogen Sensor Based on Residual Stress

**DOI:** 10.3390/s21227657

**Published:** 2021-11-18

**Authors:** Wenbo Ma, Yuyang Li, Ning Yang, Li Fan, Yanli Chen, Xuan Zhou, Jiaping Li, Caiqian Yang

**Affiliations:** 1 College of Civil Engineering and Mechanics, Xiangtan University, Xiangtan 411105, China; mawenbo@xtu.edu.cn (W.M.); 201921002151@smail.xtu.edu.cn (Y.L.); 201921002145@smail.xtu.edu.cn (Y.C.); 201921002243@smail.xtu.edu.cn (X.Z.); 201821572175@smail.xtu.edu.cn (J.L.); 2 Shandong Institute of Space Electronic Technology, Yantai 264670, China; yn3714@163.com; 3 China Academy of Space Technology, DFH Satellite Co., Ltd., Beijing 100094, China; 13810089694@139.com; 4 College of Civil Engineering, Southeast University, Nanjing 211189, China

**Keywords:** palladium film, Bragg fiber grating, durability, life and death unit method, hydrogen sensitive film

## Abstract

In this paper, in order to improve the durability of optical fiber grating hydrogen sensors, an optical fiber grating hydrogen sensor with high precision, stability, and durability is prepared. Based on the simplified two-dimensional model and finite element analysis, the effects of film thickness, coating speed, and coating times on the residual Mises equivalent stress between the sensor film and substrate were studied, and the optimum coating parameters were determined. The finite element analysis results show that the residual equivalent stress between the film and the substrate increases with the increase in the film thickness between 50 and 150 nm. The range of 200–250 nm is relatively stable, and the value is small. The coating speed has almost no effect on the residual equivalent stress. When the thickness of the film is 200 nm, the residual equivalent stress decreases with the increase in coating times, and the equivalent force is the lowest when the film is coated three times. The best coating parameters are the thickness of 200 nm, the speed of 62.5 μm/s, and the times of coating three times. The results of finite element analysis are verified by the hydrogen sensitivity test and durability test.

## 1. Introduction

Hydrogen is clean energy and an important chemical raw material. It has a wide range of applications in aerospace, fuel cell vehicles, metal smelting, and chemical synthesis [1]. Since hydrogen has the smallest molecules, hydrogen is easy to leak. When hydrogen reaches a certain content in the air (4–74.4% by volume) [2], it is easy to be ignited, which will lead to an explosion accident [3,4,5], so the detection of hydrogen concentration is very important [2]. Most of the market mature hydrogen sensors are mostly based on electrochemical principles, which makes it easy to reduce the sensor’s sensitivity by the influence of the external environment, resulting in measurement errors. This sensor easily generates electric sparks [6] during use, and the liquid hydrogen fuel used for aerospace creates a very high risk. This sensor wiring is too complicated as well, and the space is large.

The optical fiber sensor is mainly used for detection by weak light signals. It has the advantages of being intrinsically explosion-proof, small size, and lightweight, and it also has high sensitivity [7], corrosion resistance, anti-electromagnetic interference [8], and high accuracy. Therefore, the optical fiber sensor is used to detect the hydrogen concentration. It is of great significance. Hill (1978) developed a Bragg fiber grating (FBG) using the standing wave method [9]. Bulter (1994) coated a layer of palladium (Pd) film or multiple layers on the end of the fiber to form a micro-lens optical fiber hydrogen sensor [10]. Garcia (1996) and Mandelis (1998) use the difference in laser light intensity between the hydrogen-sensitive surface and the reference plate to detect hydrogen. ITO (1984) proposed Pd/tungsten trioxide (WO3) as the hydrogen-sensitive film structure. Tabib-Azar [11] (1999) plated a layer of 10–20 nm thick palladium film on the surface of the multimode optical fiber to form an evanescent wave field optical fiber hydrogen sensor; Griessen (1997) designed a guided-wave optical hydrogen sensor. Sutapun et al. [12] reported the fiber grating hydrogen sensor for the first time. Bruic et al. [13] sputtered 350 nm Pd film on FBG. Dai et al. [14] polished the FBG on the side and sputtered in the polishing area composite film. Caucheteur et al. [15] used the sol–gel method to deposit composite metal on the gate area. Arnaldo et al. [16] introduced the development of a fiber Bragg grating (FBG) sensor for the simultaneous measurement of pressure and temperature based on a polymer diaphragm. The root mean square error of pressure and sensitivity to temperature and pressure is 3.88 °C and 5.13 kPa, respectively, and the error to pressure and temperature is about 5%. Antreas et al. [17] for the first time used femtosecond laser technology to measure the relative humidity in an ultra-high performance concrete mixture using Bragg grating in a small-mode CYTOP polymer fiber (three main modes). The results show that the optical fiber sensing method in concrete structures can overcome the shortcomings of existing electronic solutions, such as scalability, reducing the single-point monitoring sensing position, and electromagnetic interference.

At present, most of the research on fiber grating hydrogen sensors pays attention to the influence of film thickness and coating material on the sensor’s performance. It ignores the influence of residual stress on the performance of the sensor. The palladium membrane itself has a hydrogen embrittlement effect, and the stress cracking caused by residual stress is easy to occur around the palladium membrane substrate in the monitoring process. In this paper, the process of fiber coating is simulated by finite element software. The effect of the coating process on the residual Mises equivalent stress between the fiber grating and palladium film system is discussed to improve the durability of the fiber grating hydrogen sensor. Based on the simplified two-dimensional model [18], we use the life-and-death element method [19]. By using this method, the effects of different film thickness, coating speed, and the number of film layers on the residual Mises between the sensor film and substrate are simulated. Then, we carry out the hydrogen sensitivity test and durability test of the sensor to verify it. Through these measures, we can get the optimal coating process. We can use the optimal coating process to make fiber grating hydrogen sensors with high precision, short response time, and stability. These sensors can make timely and accurate monitoring of the dangerous phenomenon of hydrogen leakage.

## 2. Modeling

**Figure 1:**
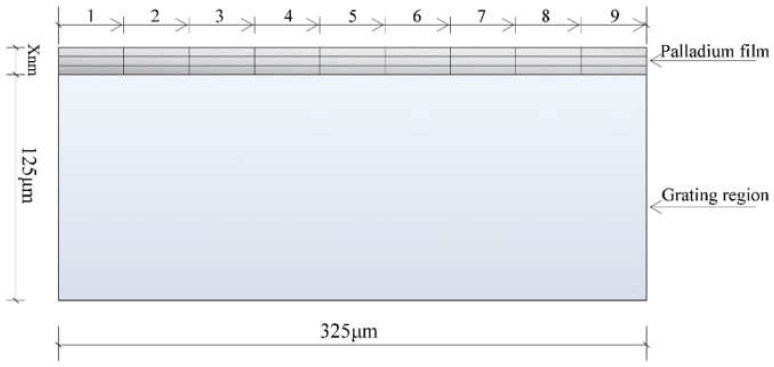
Simplified diagram of the sensor model.

The process of optical fiber coating is simulated by ANSYS software. We take the expansion of the cross-section of the fiber Bragg grating as the prototype and set it as a rectangle with a length of 375 μm and a width of 125 μm. The coating process of the specimen is simulated by simulating the gradual growth of pure palladium film at the top of the model. Before modeling, the following are assumed: (1) Regardless of the substrate’s surface roughness, the film’s upper surface is a plane. (2) In the process of finite element analysis, the physical properties of materials remain constant. (3) The materials of the thin film and substrate are isotropic [18]. Based on the hypothesis, we define a simplified two-dimensional model. The finite element method studied the effects of film thickness, coating speed, and film layers on the residual Mises equivalent stress in the film–substrate system. As shown in Figure 1, one layer of coating from 1 to 9 is recorded as one coating.

Define the element type as PLANE13; each node has two degrees of freedom, and it translates in the x and y directions of the node. PLANE13 can be used for plane stress as well as axisymmetric elements [20]. In this article, it is used to analyze plane stress. After selecting the coupling element, define the physical properties of the substrate and film materials, as shown in Table 1.

**Figure 2:**
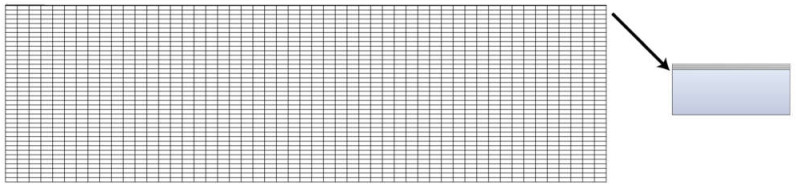
Mesh division.

The life-and-death element method was used to simulate the coating process. We simplify the sensor to a two-dimensional model to study the film thickness, coating speed, and layer number effects on the residual Mises equivalent stress in the film–substrate system. In this finite element simulation, the model is simplified to a rectangle, and the unit can be intelligently divided into quadrilateral units, as shown in Figure 2. The black area in the picture is a palladium membrane.

## 3. Numerical Simulation Results and Discussion

In order to minimize the influence of other factors on the finite element results, the data are taken from the same node. Taking the left end of the substrate–palladium film interface as the origin and the node reflected in the distance from the origin, the effect of the coating process on the residual stress between the palladium film and substrate of the hydrogen sensor was investigated. Palladium film is recorded as the induction end and fiber Bragg grating is recorded as the representation end.

### 3.1. The Influence of Coating Thickness on the Film–Substrate System

**Figure 3:**
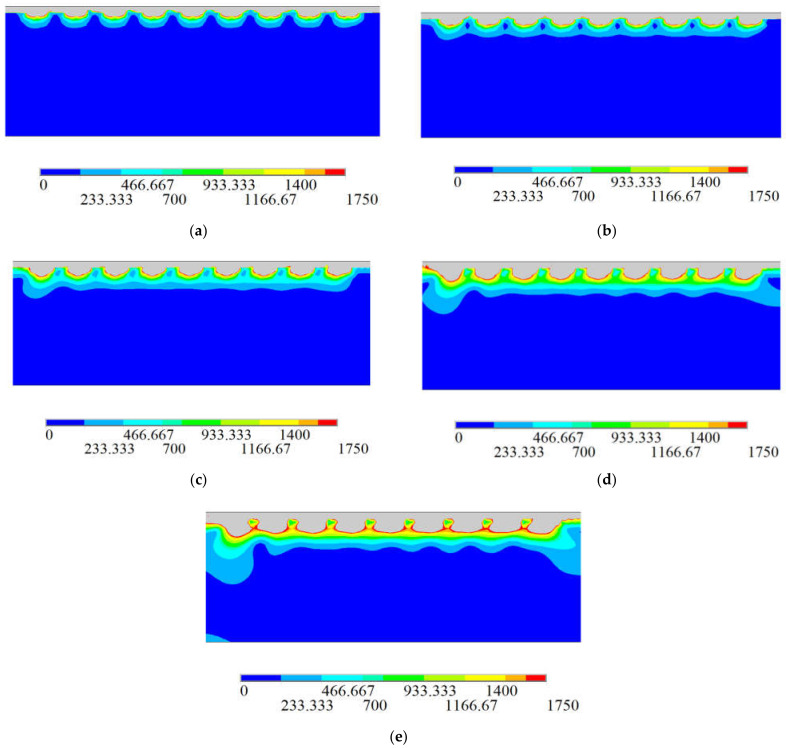
Stress cloud diagram of optical fiber under different film thickness *t*. (**a**) *t* = 60 nm; (**b**) *t* = 100 nm; (**c**) *t* = 150 nm; (**d**) *t* = 200 nm; (**e**) *t* = 250 nm.

Taking the film thickness as a variable and the time required to coat a row of cells as one as the speed, the stress cloud is shown in Figure 3. The stress distribution in the figure is approximately the same, showing a periodic change. Due to the use of the life-and-death element method for simulation, the element is gradually growing up, similar to the actual coating process. It can be concluded from the diagram that the stress range expands with the increase in film thickness *t*.

**Figure 4:**
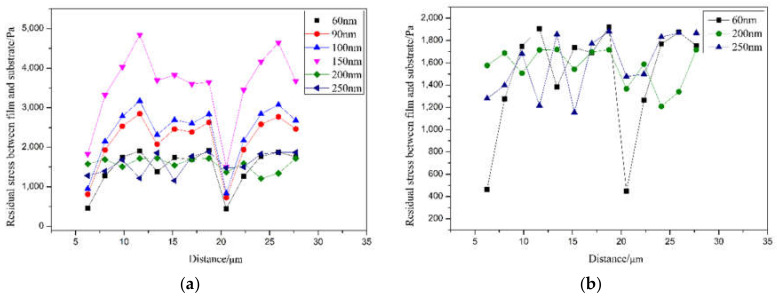
Film thickness–residual stress transformation curve. (**a**) All data; (**b**) Partial data magnification.

According to the finite element analysis results, the Mises equivalent stress change curve of the film thickness and the residual stress is shown in Figure 4. The overall stress presents a periodic fluctuation pattern. In the simulation process, the palladium film adhesion process in the actual experiment adopts the life-and-death unit method. The palladium film grows step by step, each time in the same process, resulting in periodic stress with the substrate. As shown in Figure 4a, when the thickness of the film layer is in the range of 60–150 nm, the stress value exhibits periodic variation, and the fluctuation range is large. The residual stress during the sensor preparation increases with the increase in the thickness of the film. When the thickness of the film is 150 nm, the residual stress reaches the maximum. Such stress fluctuations indicate that when the thickness of the film is between 60 and 150 nm, the residual stress value of the film and the fiber surface is very unstable. When the palladium film reacts and expands with hydrogen, the stress distribution is uneven, and the internal stress of the film is different. If it is too large, it will easily cause the film to rupture or even fall off, resulting in low durability. As shown in Figure 4b, when the thickness of the film is 200 nm and 250 nm, the fluctuation of residual stress during the preparation of the sensor is small. The residual stress between the sensor induction area and the representation area is relatively stable. At this time, the maximum residual stress is close to the minimum residual stress in numerical simulation. When the palladium membrane reacts with hydrogen to expand because the stress distribution is relatively uniform, the stresses between the various parts of the membrane during expansion are equal, so there is no rupture or shedding of the palladium membrane due to uneven internal stress. The membrane layer thickness is higher in the hydrogen sensor in the range of 200–250 nm than in the range of 60–150 nm, and the test range is large. Considering the experimental cost, the best parameter of film thickness is 200 nm, as shown in Table 2.

### 3.2. The Influence of Coating Speed on the Film–Substrate System

**Figure 5:**
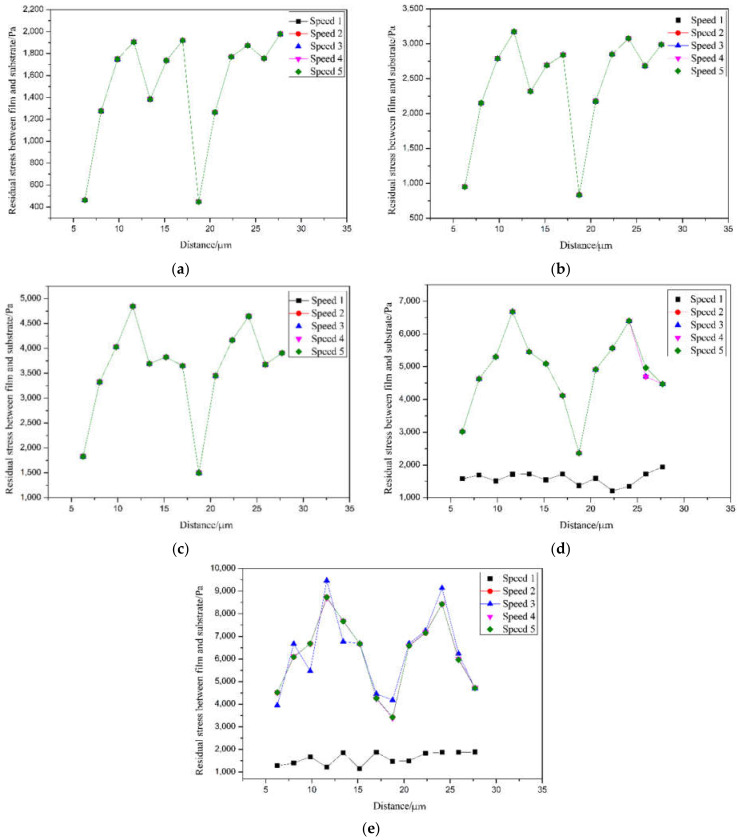
Speed–stress curve. (**a**) *t* = 60 nm; (**b**) *t* = 100 nm; (**c**) *t* = 150 nm; (**d**) *t* = 200 nm; (**e**) *t* = 250 nm.

The time required to coat a row of units is regarded as the coating speed. In the case of the same thickness, the time required to coat a row of units is divided into five groups; the time is 1/5, 2/5, 3/5, 4/5, 1, the corresponding groups are respectively speeding 62.5 μm/s, 31.25 μm/s, 20.83 μm/s, 15.625 μm/s, and 12.5 μm/s for simulation. We will mark these as speeds 1–5. According to the finite element analysis results, the change curve of the Mises equivalent stress of the coating speed and the residual stress is shown in Figure 5. When the film thickness is in the range of 60–150 nm, the coating speed has almost no effect on the fiber grating during the coating process. However, the residual stress value of the film and the optical fiber surface is unstable. When the palladium film reacts with hydrogen, the stress distribution in the film is uneven, leading to the film’s rupture or even shedding. When the thickness of the film is 200 nm and 250 nm, and the time required for the first row of the coating unit is changed from 1/5 to 2/5, that is, the coating speed is 31.25 μm/s, the residual stress increases obviously and fluctuates greatly. When the coating speed is 31.25 μm/s, the residual stress on the film’s surface and optical fiber will be unstable. When the palladium film reacts with hydrogen, the stress distribution in the film is uneven, leading to the film’s rupture or even shedding. Therefore, the best parameter of coating speed is 62.5 μm/s, as shown in Table 2.

### 3.3. The Influence of the Number of Coatings on the Film–Substrate System

**Figure 6:**
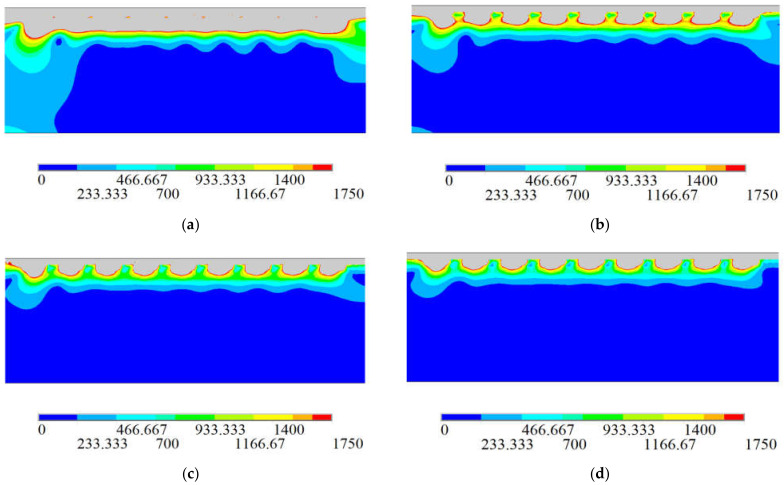
Optical fiber stress cloud diagram. (**a**) Coating one time; (**b**) Coating two times; (**c**) Coating three times; (**d**) Coating four times.

The film thickness is 200 nm, the time required to coat a row of units is one as the speed, and the film is divided into 1, 2, 3, and 4 times as variables during the coating process. The stress cloud diagram is shown in Figure 6.

**Figure 7:**
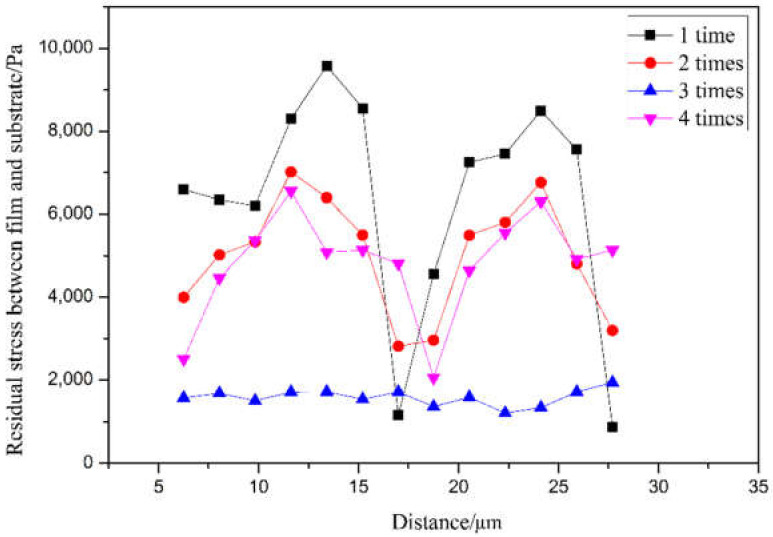
Coating layer number–residual stress equivalent stress transformation curve.

According to the finite element analysis results, the change curve of the Mises equivalent stress of the number of coating layers and the residual stress is shown in Figure 7. When the coating is one, two, and four times, the residual stress values on the film’s surface and the optical fiber are very unstable. When the palladium film reacts and expands with hydrogen, the stress distribution in the film is uneven, and the difference between the internal stresses of the film is too large. It is easy to cause the film to break or even fall off. The equivalent stress of the residual is the most stable when the coating is divided into three times, and the fluctuation range is small. When the palladium film reacts and expands with hydrogen, the stress distribution is relatively uniform, and the stress between the various parts of the film during expansion is equal. There is no palladium film rupture, or it falls off due to uneven internal stress. Under the condition that the film thickness is not changed to 200 nm, the coating layers are one, two, and four, and the stress value decreases as the number of layers increases. Therefore, the best parameter of the number of film layers is three layers, as shown in Table 2.

## 4. Experimental Verification and Result Analysis

### 4.1. Preparation of Optical Fiber Grating Hydrogen Sensor

**Figure 8:**
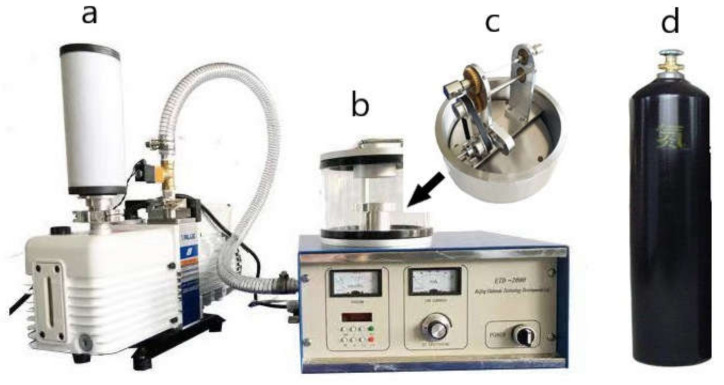
Sensor preparation system. (**a**) Vacuum pump; (**b**) Magnetron sputtering apparatus; (**c**) Optical fiber rotary table; (**d**) Nitrogen bottle.

**Figure 9:**
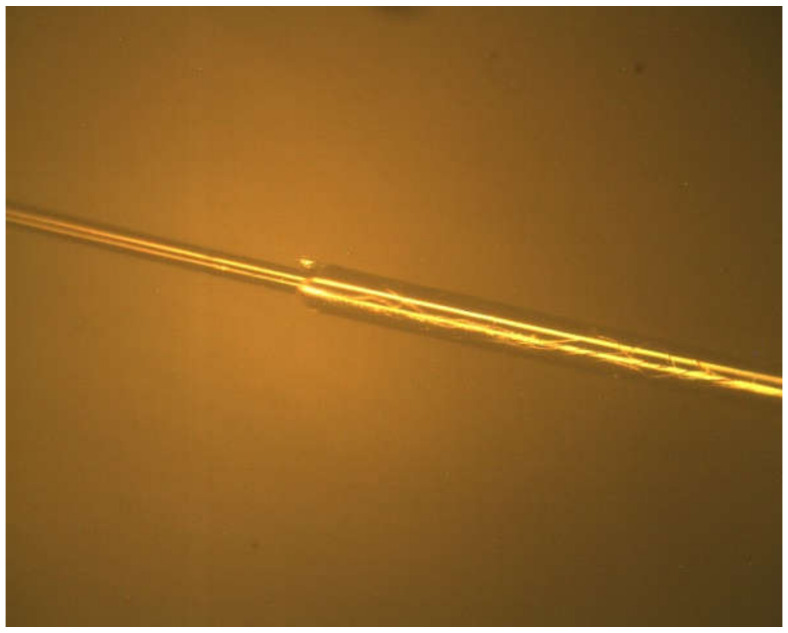
Palladium film coating under the crystal phase microscope.

In order to verify the correctness of the finite element results, the hydrogen sensitivity test and durability test of the sensor were carried out. First of all, the sensor is prepared. We use a self-made rotary table and a magnetron sputtering instrument to form a preparation system, as shown in Figure 8. The function of the turntable is to ensure the uniform rotation of the fiber grating during the coating process, make the Pd particles uniformly adhere to the gate area, and reduce the residual stress between the palladium film and the gate and improve the durability of the sensor. We use anhydrous ethanol to clean the surface of fiber Bragg grating and dry it naturally. We use the turntable fixture to fix the fiber grating and connect the turntable with the magnetron sputtering instrument. Then, we turn on the vacuum pump, and when the vacuum gauge reads 4 × 10^−2^ mbar, we pass in nitrogen as a protective gas to prevent the sensor from failing during the preparation process [21]. When the vacuum gauge reads 10 Pa, we turn the current knob to start sputtering. The coating thickness and coating speed can be controlled by the coating time and current value; the current knob can control the coating times. The FBG fiber grid area was coated. According to the finite element analysis results, the film thickness was set to 50, 100, 150, and 200 nm, the coating speed was 62.5 μm/s, and the number of coatings was three times. Due to the physical properties of palladium, the palladium membrane is brittle and easy to break or fall off. The surface of the coating process will be observed using a microscope to see if the film layer is uniform, whether a rupture or falling off, as shown in Figure 9. The left end is the grid area, that is, the coating area, the surface of the grid area is smooth, and the palladium film is evenly distributed on the surface of the grid area, without cracks and shedding parts. The right end is a non-grid region, some of which are within the sputtering range of the magnetron sputtering instrument, and there are also some palladium films. There are obvious cracks in the palladium film in the non-gate region, which may be due to the uneven distribution of the film due to the unsmooth interface between the gate region and the non-gate region.

**Figure 10:**
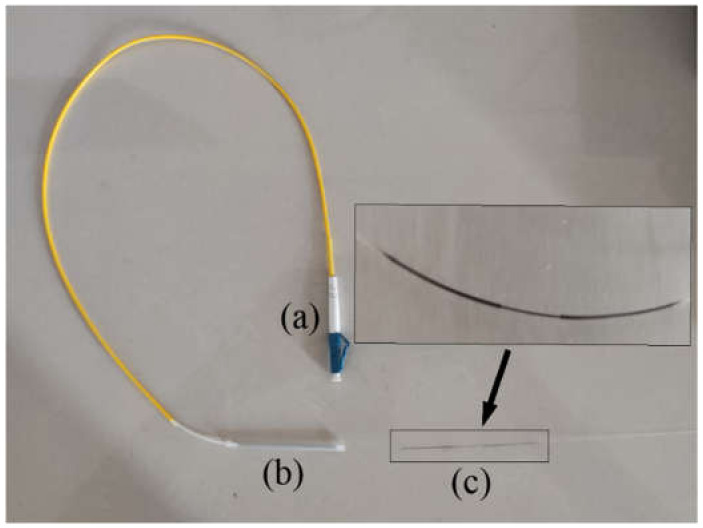
Optical fiber grating hydrogen sensor. (**a**) LC jumper; (**b**) Heat shrink tube; (**c**) Hydrogen-sensitive probe.

After the fiber sputtering was completed, we cut the end of the fiber grating, cleaned it with anhydrous ethanol, and dried it naturally. Then, we peel off the protective layer of the LC jumper’s tail end, cut the jumper’s tail end with an optical fiber cutter, and then clean the tail fiber surface with anhydrous ethanol. We use a welding machine to weld the fiber Bragg grating and the jumper, then use a heat-shrinkable tube to protect the connection, and finally form a complete fiber Bragg grating hydrogen sensor, as shown in Figure 10.

### 4.2. Hydrogen Sensitivity Test and Durability Test

**Figure 11:**
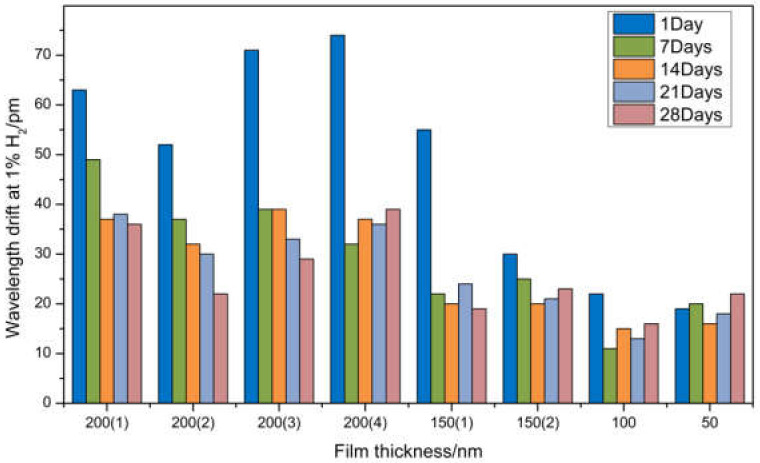
Wavelength drift of hydrogen sensors with different thicknesses at 1% H_2_.

We put the hydrogen sensor in the gas chamber, observe the original central wavelength of the optical fiber, and then turn on the gas tank switch. We choose the appropriate pressure to release hydrogen to ensure that the slow flow rate does not affect the sensor to measure the concentration of hydrogen. When the volume fraction of hydrogen in the gas chamber reaches 1%, the change of the central wavelength of the optical fiber is observed after waiting for a period of time. When the central wavelength of the optical fiber reaches the maximum, the maximum value of the central wavelength is calculated with the original central wavelength, and the drift of the optical fiber wavelength is obtained when the volume fraction of hydrogen is 1% The durability experiments were carried out at 7 days, 14 days, 21 days, and 28 days after the first measurement. There were four samples in each group, and the data were processed as average. The wavelength shift is shown in Figure 11. The first time the sensor wavelength drift is tested, the volume fraction of hydrogen is 1%. It can be seen that the wavelength drift of the sensor with a palladium film thickness of 200 nm is above 50 pm. Compared with other self-reference optical structures [22,23], the sensor performance is relatively stable. The 50–150 nm sensor does not achieve the expected effect in monitoring the hydrogen concentration, and its performance is unstable. The thickness of the sensor’s palladium film is 200–50 nm, and the wavelength drift decreases with the decrease in the thickness, which is consistent with the simulation results. The second measurement was carried out a week later, and the wavelength drift was generally reduced. The reason was that the palladium film was brittle and swelled after reacting with hydrogen. As a result, the expansion of the second experiment was not enough to cause a large change in the grating region, and the drift was reduced. When the third measurement was carried out 14 days later, the wavelength drift of the sensor changed, but the fluctuation was small. When the third measurement is carried out 14 days after the first measurement, the wavelength drifts of the sensor change, but the fluctuation is small. After the fourth and fifth measurements, it is found that the wavelength drift is relatively stable at a certain value. This is due to the rupture of part of the palladium membrane caused by three hydrogen sensitivity tests, which release the residual stress during the preparation of the sensor. However, because the residual stress in the preparation process of the sensor is small, and there are still some residual thin films, it still has a certain ability to detect hydrogen.

**Figure 12:**
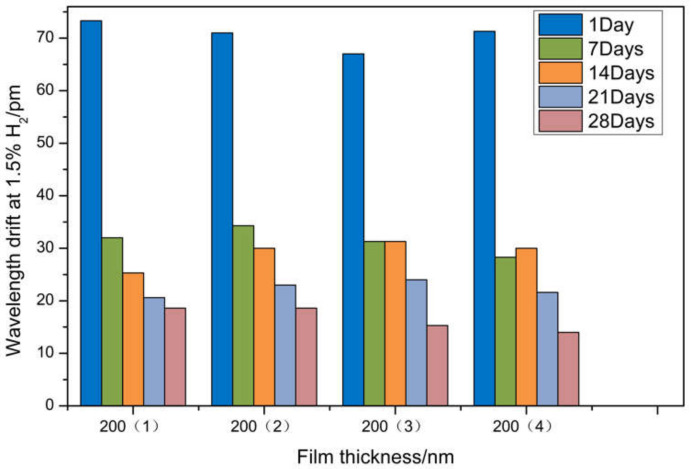
Wavelength drift of hydrogen sensors with different thicknesses at 1.5% H_2_.

It can be seen from Figure 11 that the hydrogen sensor with the film thickness of 200 nm, coating speed of 62.5 μm/s, and coating times of three times has the best durability and stable performance. Therefore, the sensor with a film thickness of 200 nm is selected as the test sensor in the follow-up experiment. In order to improve the accuracy of the experimental data, a new sensor was prepared. When the volume fraction of hydrogen in the gas chamber is 1.5%, we test and record with the same test method. The experiment was divided into four groups with three samples in each group, and the data were processed as average. The wavelength shift is shown in Figure 12. In the first test, the wavelength drift was above 65 pm, and the drift under 1% hydrogen concentration increased but did not reach the 50 pm/% growth rate. The reason is that the relationship between the expansion rate of palladium membrane and hydrogen concentration of 200 nm thickness is non-linear, and the drift change rate caused by palladium membrane expansion decreases. When the second measurement was carried out a week later, the wavelength drift generally decreased. The drift of the sensor is reduced by more than 1% of the concentration of hydrogen. The reason is that the high concentration of hydrogen is more destructive to the sensor, and the reduction of residual stress in the coating process is not enough to make up for the hydrogen embrittlement effect of palladium film. When the third measurement was carried out 14 days later, the wavelength drift of the sensor changed, but the fluctuation was small. The fourth and fifth measurements were carried out after 21 days and 28 days, respectively, and it was found that the wavelength drift was relatively stable at a certain value. This is due to the rupture of part of the palladium membrane caused by three hydrogen sensitivity tests, which release the residual stress during the preparation of the sensor. However, because the residual stress in the preparation process of the sensor is small, and there are still some residual thin films, it still has a certain ability to detect hydrogen.

**Figure 13:**
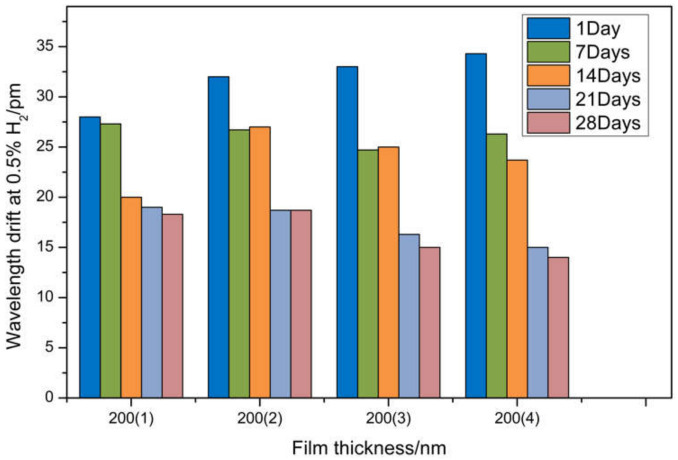
Wavelength drift of hydrogen sensors with different thicknesses at 0.5% H_2_.

In order to improve the accuracy of experimental data, a new sensor was prepared. When the volume fraction of hydrogen in the gas chamber is 1.5%, we test and record with the same test method. The experiment was divided into four groups with three samples in each group, and the data were processed as average. The wavelength shift is shown in Figure 13. The wavelength shift of the first test is all above 25 pm, with a growth rate of more than 50 pm/%. The reason is that the relationship between the palladium membrane expansion rate of 200 nm thickness and hydrogen concentration is non-linear, and the drift change rate caused by palladium membrane expansion is greater than that under 1% concentration of hydrogen. After 7 days and 14 days of measurement, it is found that the wavelength drift of the sensor changes little, and the durability is improved compared with other self-reference optical structures. It is proved that the optimization of process parameters can significantly reduce the residual stress in the coating process and improve the durability of the sensor. The fourth and fifth measurements were carried out after 21 days and 28 days, respectively, and it was found that the wavelength drift was relatively stable at a certain value.

## 5. Conclusions

The film thickness and coating times influence the residual Mises equivalent stress. According to the finite element analysis results, the residual equivalent stress in the film and the substrate system is proportional to the film thickness between 50 and 150 nm; between 200 and 250 nm, the value of residual equivalent stress decreases and is relatively stable. Therefore, 200 nm is more suitable for preparing a film–substrate system when only considering the film thickness. The residual equivalent stress in the film–substrate system is inversely proportional to the number of coatings. When the number of coatings is 3, the value of the residual equivalent stress is the smallest. To sum up, the best coating process parameters are as follows: the thickness of the film is 200 nm, the coating times are three times, and the coating speed of 62.5 μm/s.

Based on finite element analysis, the sensor’s hydrogen sensitivity test and durability test were carried out. According to the hydrogen sensitivity test results, when the hydrogen concentration is 1%, the first hydrogen sensitivity test, the wavelength shift of the sensors with the palladium film thickness of 200 nm is above 50 pm. The sensor with 150 nm film thickness fluctuates greatly, and its performance is unstable. The thickness of the palladium film is 50–200 nm, and the wavelength drift increases with the increase in the thickness, which is consistent with the simulation results. The durability test shows that the wavelength drift of the sensor decreases over time until it is stable near a certain value, and the sensor with 200 nm film thickness has the best durability.

On this basis, hydrogen sensitivity and durability tests were carried out at 0.5% and 1.5% concentrations. According to the experimental results, the relationship between wavelength drift and palladium film thickness is non-linear, and the sensor has the best sensitivity and durability at 0.5% concentration. The optimization of the coating process parameters at 1.5% concentration is not enough to make up for the hydrogen embrittlement effect of palladium metal. Therefore, the best test range of fiber grating hydrogen sensors is 0.5–1% hydrogen concentration.

## Figures and Tables

**Table 1:** Material parameters.

Material	Elastic Modulus (GPa)	Poisson’s Ratio	Density $\left(g/cm^{3}\right)$	Specific Heat Capacity (J/kg⋅°C)	Thermal Expansion Coefficient (/K)	Thermal Conductivity (w/cm⋅K)
Pd	121	0.39	12.02	244	11.2$\;\times \;10^{-6}$	71.8
SiO_2_	73.1	0.16	2.2	966	0.5$\;\times \;10^{-6}$	0.27

**Table 2:** Optimum coating process parameters.

Film Thickness/nm	Coating Speed/(μm/s)	Coating Times
200	62.5	3

## Data Availability

The data in this article can be obtained from the corresponding author.
